# Parathyroid Hormone (1-34) Might Not Improve Early Bone Healing after Sinus Augmentation in Healthy Rabbits

**DOI:** 10.1155/2017/6087676

**Published:** 2017-02-09

**Authors:** Jisun Huh, Ui-Won Jung, Kyeong-Mee Park, Hyun Sil Kim, Kee-Deog Kim, Wonse Park

**Affiliations:** ^1^Department of Advanced General Dentistry, College of Dentistry, Yonsei University, Seoul, Republic of Korea; ^2^Department of Periodontology, College of Dentistry, Yonsei University, Seoul, Republic of Korea; ^3^Department of Oral Pathology, College of Dentistry, Yonsei University, Seoul, Republic of Korea

## Abstract

*Purpose.* This study evaluated the effect of administering intermittent parathyroid hormone [PTH (1-34), henceforth PTH] on the early-stage bone healing of maxillary sinus augmentation in healthy rabbits.* Materials and Methods.* Bovine bone mineral was grafted on the sinuses of 20 female New Zealand white rabbits. The animals were randomly divided into two groups, PTH (*n* = 10) or saline (*n* = 10), in which either PTH or saline was injected subcutaneously 5 days a week for 2 weeks. Half of the animals in each group were killed at 2 weeks postoperatively and the other half were killed at 4 weeks postoperatively. The dosage of PTH was 10 *μ*g/kg/day. Radiographic and histomorphometric analyses were performed.* Result.* The new bone area (NBA) did not differ significantly between the PTH and saline groups. The NBA in the PTH group in the total augmented area and in the demarcated window, center, and Schneiderian membrane regions increased significantly from 2 to 4 weeks. The number of osteoclasts decreased significantly from 2 to 4 weeks in both groups, with no difference between the two groups.* Conclusion.* Intermittent PTH might not stimulate new bone formation in healthy rabbits during the first 4 weeks of healing.

## 1. Introduction

Teriparatide, a portion of recombinant human parathyroid hormone [PTH (1-34), henceforth PTH], is an osteoanabolic agent that was approved for osteoporosis treatment by the United States Food and Drug Administration in 2002 [1, 2]. It was reported first in 1923 that parathyroid extract stimulated new bone formation contrary to the original function, calcium release from bone tissue [3], and PTH is known to have osteocatabolic effect when infused continuously and osteoanabolic effect when administrated intermittently [4]. PTH stimulates new bone formation by increasing the number of osteoblasts and decreasing the apoptosis of osteoblasts when it is administrated intermittently [5]. PTH has uniqueness in being a real anabolic agent, while osteoporosis medicaments that have been used are antiresorptive agents [6]. The anabolic effect has been applied in diverse applications beyond osteoporosis treatment, and many animal and clinical studies have found that it decreased the fracture risk, stimulated fracture healing, increased the bone density, and exerted other positive effects [7–9]. In the dental field it was reported that PTH stimulated periodontal regeneration [10], implant healing [11], healing of medication-related osteonecrosis of the jaw [12, 13], and orthodontic movement [14].

Several studies have investigated the effects of PTH on bone graft. The early trials involved spinal fusion surgery, with PTH showing positive effects on the survival of allografts and autografts [15–17]. In other animal studies, PTH stimulated distraction osteogenesis in rabbits [18] and allograft integration in calvarial bone defects in mice [19]. Animal studies related to dental effects found that PTH stimulated healing after bone graft on extraction sockets [20] and calvarial bone autografts on the mandible in rats [21]. However, studies of the effect of PTH on bone grafts are still deficient, and PTH has not been evaluated previously in sinus augmentation.

Maxillary sinus grafting via a lateral window approach is a widely performed operation for vertical bone deficiency due to maxillary sinus pneumatization. It is the grafting procedure of bone substitute material into maxillary sinus in order to obtain adequate bone height for placement of dental implants. It has the limitation of a long healing period of about 6 months [22]. If PTH stimulates the bone healing of sinus augmentation, total treatment period of the implant restoration for posterior maxillary dentition with lack of bone height could be reduced and so the masticatory function of these patients could be restored earlier.

The rabbit sinus model is a common animal model used in studies of sinus augmentation. The air pressure in the maxillary sinus is similar and the Schneiderian membrane lining is the same in rabbits and humans, and rabbits are both easy to care for and inexpensive [23, 24]. The metabolic rate is three to four times faster in rabbits than in humans, and the effects on bone healing can be confirmed within weeks [25]. Several studies using this model have shown differences between control and experimental groups at healing periods of 2 and 4 weeks [26–28].

Based on previous studies that were mentioned above, it is expected that intermittent PTH could stimulate the bone regeneration after maxillary sinus augmentation. Several studies performed in maxillofacial region of rabbit or rat showed especially that PTH increased jaw mineral density in rabbit [29], increased volume of calvarial autograft on mandible in rat [21], and increased bone fill of graft on extraction socket in rat [20]. These might support our hypothesis.

The present study is the first trial to investigate the effects of PTH on sinus augmentation and we evaluated the effect of intermittent PTH administration on early bone healing in sinus augmentation using healthy rabbits.

## 2. Materials and Methods

### 2.1. Animals

Twenty female New Zealand white rabbits weighing 2.8–3.2 kg were used. Animals were cared for under standard laboratory conditions with free access to water and a standard laboratory pellet diet. The selection, management, and preparation of animal followed the protocol of the Institutional Animal Care and Use Committee of Yonsei Medical Center, Seoul, Republic of Korea (the ethics approval number was 2012-0224).

### 2.2. Experimental Materials

#### 2.2.1. PTH

PTH (Forsteo, Eli Lilly, Houten, Netherlands) was used to stimulate bone healing. Each 80 *μ*L of injection solution contained 20 *μ*g of PTH. Based on previous studies, PTH was applied at a dose of 10 *μ*g/kg, with the amount injected calculated according to the concentration of PTH and the animal's weight.

#### 2.2.2. Deproteinized Bovine Bone Mineral

Deproteinized bovine bone mineral (Bio-Oss, Geistlich Pharma, Wolhusen, Switzerland) was used as a bone substitute material for maxillary sinus augmentation. The particle size was 250–1000 *μ*m and 0.15 g was grafted per sinus.

### 2.3. Experimental Design

In the PTH group (*n* = 10), PTH was injected 5 days a week for 2 weeks, for a total of nine times (except on the operation day). The dosage of PTH was 10 *μ*g/kg/day. The animals were killed at 2 weeks (*n* = 5) or 4 weeks (*n* = 5) postoperatively. Animals in the saline group (*n* = 10) were injected with the same amount of saline on the same schedule as in the PTH group, and they were also killed at 2 weeks (*n* = 5) or 4 weeks (*n* = 5) postoperatively.

### 2.4. Surgical Procedure

The overall surgical procedure used in this study followed that used by Choi et al. [23]. A mixture of ketamine hydrochloride (Ketalar, Yuhan, Seoul, Republic of Korea) and xylazine (Rompun, Bayer Korea, Seoul, Republic of Korea) was injected intramuscularly for general anesthesia. After shaving the nasal bone area and preparing the skin surface with povidone iodine, 2% lidocaine (Lidocaine HCl, Huons, Seoul, Republic of Korea) was injected for local anesthesia. A linear incision was made along the sagittal midline on the nasal bone, and then a full-thickness flap was elevated bilaterally. On the lining bony plate over the maxillary sinus area at a position decided in the previous study [23], a circular bony window with a diameter of 6 mm was made using a trephine bur (3i Implant Innovation, Palm Beach Gardens, FL, USA) under saline irrigation while taking great care not to perforate the Schneiderian membrane. The excised bony disc was removed, and then the Schneiderian membrane was elevated carefully and 0.15 g of deproteinized bovine bone mineral was grafted. The flap was replaced and sutured layer by layer with 4-0 absorbable glyconate monofilament (Monosyn, B-Braun, Aesculap, Center Valley, PA, USA). The stitches were removed 7 days postoperatively.

### 2.5. Radiographic Analysis: Microcomputed Tomography

**Figure 1:**
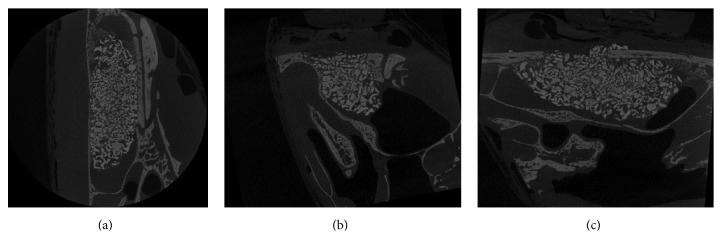
Microcomputed tomography (microCT) analysis. Axial, coronal, and sagittal views ((a) to (c)).

Block sections that included the maxillary sinus and surrounding tissue were excised and fixed in 10% formalin solution for 10 days. Microcomputed tomography (microCT) images of the fixed specimens were obtained using high-resolution microCT (Skyscan 1173, Bruker, Kontich, Belgium) at a resolution of 35 *μ*m (achieved using 130 kV and 60 *μ*A). The images were stored in Digital Imaging Communication in Medicine format (Figure 1). A region of interest (ROI) that included all grafted material and newly formed tissue was selected, and the total augmented volume was measured. A grayscale threshold range of 58~255 was applied when calculating the total mineralized volume (TMV) within each ROI.

### 2.6. Histomorphometric Analysis

After microCT scanning, the block specimens were decalcified using a decalcification solution (Rapid Cal Immuno, BBC Biochemical, Mt Vernon, WA, USA), embedded in paraffin, and sectioned serially at 5 *μ*m in the coronal direction. The two center-most sections were selected: one was stained with Masson's trichrome and the other was stained with hematoxylin-eosin. Images were obtained with the aid of a light microscope (Leica DM 2500, Leica Microsystems, Wetzlar, Germany, and Virtual Microscope VS120, Olympus Corporation, Tokyo, Japan), and they were analyzed histomorphometrically by a masked experienced observer (K.P.) using PC-based image-processing software (Adobe Photoshop CS4, Adobe Systems, San Jose, CA, USA).

**Figure 2:**
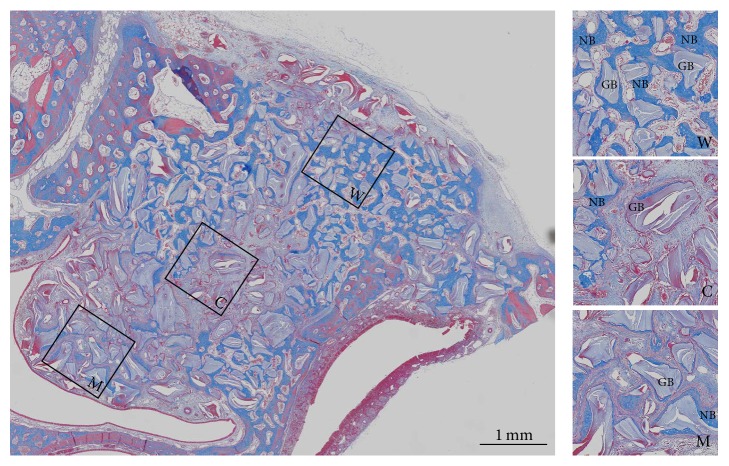
Masson's-trichrome-stained slide with three squares showing enlarged views in the window (W), center (C), and Schneiderian membrane (M) regions. The particles stained light purple are grafted bone substitute material, and new bone is stained blue. NB: new bone; GB: grafted bone substitute.

The boundary of the total augmented area (TAA) was traced in slides stained with Masson's trichrome. Square window, center, and Schneiderian membrane regions (1 mm × 1 mm) were selected within the TAA in each slide (Figure 2). The new bone area (NBA) was measured in the TAA, window, center, and Schneiderian membrane regions, and the percentage of NBA to TAA was calculated.

**Figure 3:**
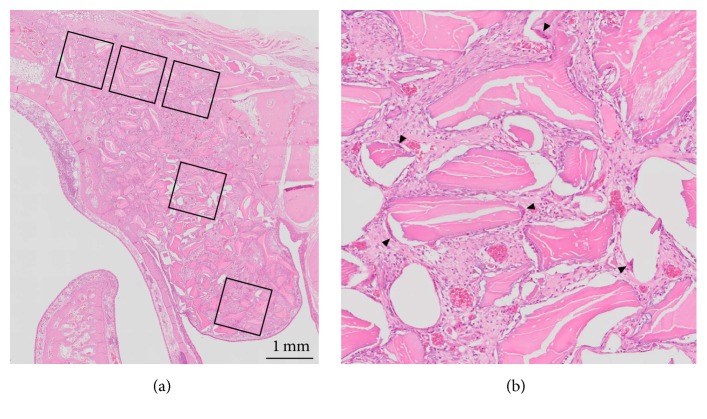
Hematoxylin-eosin-stained slide showing the five regions selected for osteoclast counting (a). Magnified image of the center region (b). Multinucleated cells indicated by black arrowheads are osteoclasts.

In slides stained with hematoxylin-eosin, five square regions (also 1 mm × 1 mm) were selected in each animal (Figure 3). The osteoclasts (which are multinucleated giant cells) were counted within the five regions, and the total number of osteoclasts in the five regions in each slide was obtained.

### 2.7. Statistics

Sample size was calculated based on three previous studies in rabbits. Mashiba et al. reported that the bone mineral density in tibia was 0.25 ± 0.004 g/cm^2^ in saline vehicle injected group and 0.27 ± 0.009 g/cm^2^ in PTH injected group [30]. Calculated sample size from these data under 80% of power level and alpha level 0.5 was three per group. The other two studies were performed in rabbit model of osteoporosis. Almagro et al.'s study of tibial implant represented that bone area % in peri-implant trabecular bone of PTH and vehicle injected group was 19.0 ± 6.4 and 11.6 ± 3.7, respectively [31]. Bone density in mandible reported by Bellido et al. was 0.231 ± 0.004 g/cm^2^ in PTH injected group and 0.224 ± 0.003 g/cm^2^ in saline injected group [29]. Sample size calculated from these two studies under same power and alpha levels was five per group.

Data were analyzed using SPSS (version 23.0, IBM Corporation, Armonk, NY, USA). The Mann–Whitney test was used to compare each parameter between the saline and PTH groups and to compare between 2 and 4 weeks of healing in the saline and PTH groups. The Friedman test and the Wilcoxon test were used to assess differences in NBA values between the window, center, and Schneiderian membrane regions within each group. The cutoff for statistical significance was set at *p* < 0.05, with Bonferroni correction being applied.

## 3. Results

### 3.1. Clinical Findings

The Schneiderian membrane was perforated in four sinuses during the operations: two in the saline group and two in the PTH group. All of these perforations were minor (smaller than 1 mm) and three of them healed well. However, one animal in the saline group suffered from maxillary sinusitis during the healing period, and severe pus was observed in the sinus when the animal was killed at 2 weeks. This animal was therefore excluded from the analysis.

### 3.2. Radiographic Analysis: MicroCT

**Figure 4:**
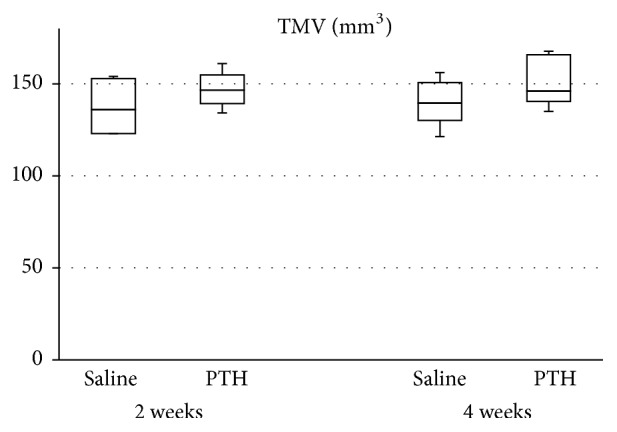
Total mineralized volume (TMV) in the total augmented region as determined by microCT analysis. There were no significant differences between the saline and PTH groups at 2 and 4 weeks. Each boxplot shows the median, first, and third quartiles and range.

The volumetric analysis results for microCT are displayed in Figure 4. TMV did not differ significantly between the saline and PTH groups at either 2 or 4 weeks.

### 3.3. Histological Findings

The histological healing pattern did not appear to differ between the saline and PTH groups.

At 2 weeks, both the saline and PTH groups showed newly formed bone along the lateral wall in addition to the existing bone present mainly in the window area. New bone formation started from the original bone surface and incorporated bone substitute particles. In some animals, thin new bone surrounding bone substitute particles was observed near the Schneiderian membrane.

At 4 weeks in both groups, all of the animals except for one in the saline group exhibited progressive new bone formation. The new bone was evenly distributed in the entire window and also within the middle region, extending from lateral to center, and was advanced compared to the situation at 2 weeks. Newly formed bone, bone substitute material, and existing bone were densely interconnected as if they had originally been in the same body.

### 3.4. Histomorphometric Analysis

**Figure 5:**
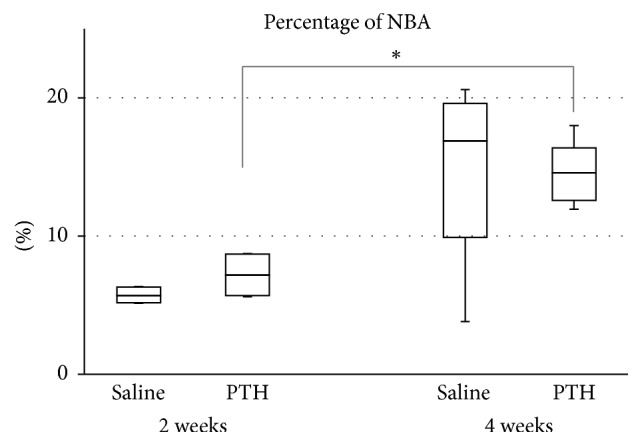
Percentage of new bone area (NBA) relative to the total augmented area. ^*∗*^Significant increase from 2 to 4 weeks in the PTH group (*p* = 0.009).

The percentage of NBA did not differ between the PTH and saline groups at either 2 or 4 weeks [2 weeks: saline group, 5.72 ± 0.62% (mean ± SD); PTH group, 7.19 ± 1.50%; *p* = 0.142; 4 weeks: saline group, 15.18 ± 6.59%; PTH group, 14.50 ± 2.28%; *p* = 0.779]. NBA was larger at 4 weeks than at 2 weeks in the PTH group (*p* = 0.009) but did not differ significantly between these two time points in the saline group (*p* = 0.251) (Figure 5). At 4 weeks, the variation among individual animals was smaller in the PTH group than in the saline group.

**Figure 6:**
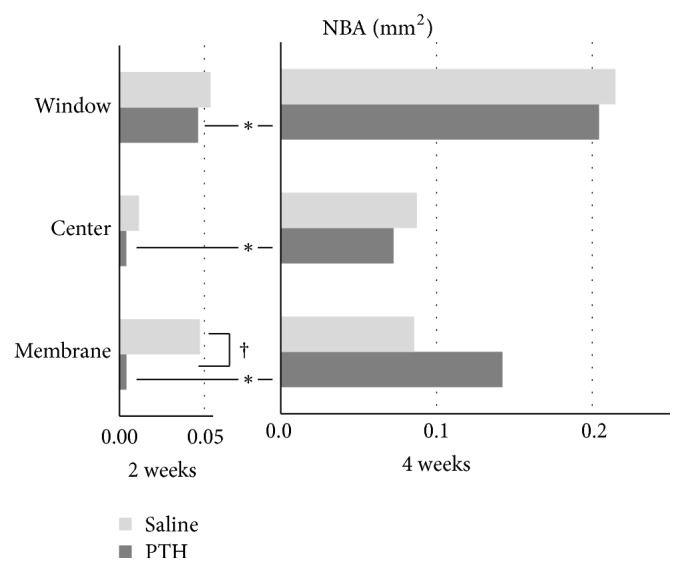
NBA within 1-mm^2^ squares in the window, center, and Schneiderian membrane regions. ^*∗*^Significantly different between 2 and 4 weeks within the PTH group (*p* = 0.009, 0.024, and 0.015 in the window, center, and Schneiderian membrane regions, resp.). ^†^Significantly different between the saline and PTH groups in the Schneiderian membrane region at 2 weeks (*p* = 0.046).

Figure 6 shows the NBA values in the window, center, and Schneiderian membrane regions. At 2 weeks, NBA was larger in the saline group than in the PTH group in the Schneiderian membrane region being statistically significant (saline group, 0.047 ± 0.051 mm^2^; PTH group, 0.004 ± 0.006 mm^2^; *p* = 0.046). The NBA values in the window, center, and Schneiderian membrane regions were significantly larger at 4 weeks than at 2 weeks in the PTH groups but did not differ significantly between these two time points in the saline group. When comparing between different regions, there were no significant differences at either 2 or 4 weeks in both the saline and PTH groups.

**Figure 7:**
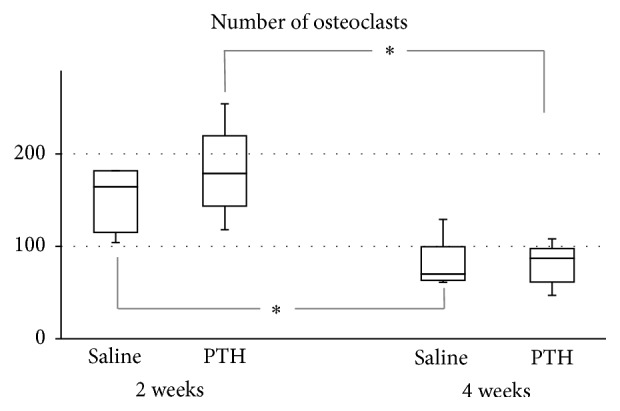
Numbers of osteoclasts. ^*∗*^Significantly decreased from 2 to 4 weeks in both the saline and PTH groups.

The number of osteoclasts decreased significantly from 2 to 4 weeks in both the saline and PTH groups [saline group: 2 weeks, 153.8 ± 36.7, 4 weeks, 79.0 ± 28.2; *p* = 0.027; PTH group: 2 weeks, 181.0 ± 48.6, 4 weeks, 81.0 ± 22.3; *p* = 0.009] (Figure 7). They did not differ significantly between the saline and PTH groups at either 2 or 4 weeks.

## 4. Discussion

This study evaluated the effect of administering intermittent PTH on early bone healing of sinus augmentation in a healthy rabbit sinus model. The amount of new bone formation and number of osteoclasts did not differ between the PTH and saline groups at both 2 and 4 weeks. NBA increased significantly from 2 to 4 weeks in the PTH group, with this group being characterized by relatively small variations in NBA values among the individual animals.

The dose and injection interval of PTH used in the present study followed those in previous studies. Studies involving rabbits have used PTH doses of 6~40 *μ*g/kg and injection schedules varying from three times a week to daily. The most common values were 10 *μ*g/kg and five days a week or daily administration [30–32], and so we used a dose of 10 *μ*g/kg and a schedule of five days a week.

MicroCT analysis was used for confirming the proper retention of bone substitute material within the maxillary sinus. The mineralization of the newly formed bone was partly observed in some histologic section and the new bone was almost under mineralized state, which could not be detected by microCT. Same level of TMV in the saline and PTH groups at 2 and 4 weeks means that the bone substitute material was retained well through the healing period up to 4 weeks. The quantity of the newly formed bone was measured in Masson's trichrome stained histologic section. Special method for distinguishing between the new and preexisting bone was not necessary because the bone substitute material was grafted into maxillary sinus, which is empty space without preexisting bone. NBA values were measured in 2-dimensional histologic sections so the total augmented area could vary according to the shape of sinus and total augmented volume. Percentage of NBA is the proportion of NBA to TAA in the histologic section so it is a more reliable value.

The numbers of osteoclasts were counted to verify the stimulation of bone remodeling by PTH. The extension of the bone remodeling space as determined by the number and activity of osteoclasts is important for confirming an osteoanabolic effect of PTH, in addition to increasing the number of osteoblasts [33]. Osteoclasts are multinucleated giant cells that are easily countable, in contrast to osteoblasts having shapes similar to fibroblasts at the early stage of new bone formation. The osteoclast population was consistent with the NBA values, but there was no significant difference between the two study groups.

Data on the osteoanabolic effects of intermittent PTH in animal studies are accumulating [4]. When reviewing studies limited to rabbits, most studies have found positive effects of intermittent PTH on implant osteogenesis [11, 31, 34] and distraction osteogenesis [18, 35]. A search for studies involving other animals and other sites did not reveal results of intermittent PTH administration not enhancing bone healing.

Several factors could be considered when explaining no differences of NBA and the osteoclast population between the saline and PTH groups. The first factor is that young and healthy animals were used in this study. In healthy animals, the normal regulation system for maintaining homeostasis is working well and any temporary increase in the PTH level such as that induced by the administration of PTH could be cancelled by the homeostasis system. PTH is an osteoanabolic agent for osteoporosis patients [1, 2], and it may affect sinus augmentation in individuals who have diseases that compromised bone.

The second factor is that PTH is one of the systemic factors related to bone metabolism. Other systemic factors are estrogen, progesterone, and cortisol, and there are also several local factors [36]. The differentiation and activation of osteoblasts and osteoclasts are regulated by interactions between these factors, and so the effect of PTH on bone could be offset by the action of other related factors.

The third factor is the site specificity of PTH. Qi et al. reported that lumbar vertebra represented total restoration of lost cancellous bone and only marginal restoration was observed in proximal tibia in ovariectomized rat [37]. Several studies showed that the responsiveness to PTH varies according to skeletal sites in human [38, 39]. Most studies in rabbit were performed in tibia or lumbar spine and PTH have not been evaluated previously in maxillary sinus.

Last possible explanation is that the penetration rate into sinus mucosa varies according to medicines. It is supported by a study that showed that amoxicillin and clavulanic acid had different penetration rate into sinus mucosa [40]. There was no study that revealed the level of systemically administrated PTH in sinus mucosa. The results of the present study can make it possible to speculate low penetration rate of PTH into sinus mucosa. Jung et al. reported that locally applied PTH stimulated bone regeneration on peri-implant bone defect in dog and calvarial bone defect in rabbit [41, 42]. Local application of PTH can be alternative to systemic administration on sinus augmentation.

However, meaningful results were obtained in the PTH group, with a significant increase in new bone formation from 2 to 4 weeks and with smaller deviations in NBA values among the individual animals. These results indicate that PTH could enhance bone formation after the early healing period and also increase the consistency of bone healing.

This study was performed in young healthy rabbits, and so it was not possible to confirm whether intermittent PTH exerts osteoanabolic effect in individuals with diseases that compromised bone. This could be evaluated in further studies involving aged animals with osteoporosis induced by ovariectomy and longer healing periods.

## 5. Conclusion

Within the limitations of this study, it is concluded that intermittent PTH might not stimulate new bone formation in healthy rabbits for up to 4 weeks of healing. The clinical relevance of this study is that PTH could increase the predictability of the sinus augmentation procedure, although greater new bone formation might not be expected if PTH is used as a supplement treatment in sinus augmentation in healthy individuals.
